# Uterus Wrapping: A Novel Concept in the Management of Uterine Atony during Cesarean Delivery

**DOI:** 10.1155/2015/195696

**Published:** 2015-08-26

**Authors:** N. Kimmich, W. Engel, M. Kreft, R. Zimmermann

**Affiliations:** Division of Obstetrics, University Hospital of Zurich, Frauenklinikstrasse 10, 8006 Zurich, Switzerland

## Abstract

Uterine atony during cesarean delivery is a serious cause of maternal morbidity and mortality. Management strategies include medical treatment with uterotonic agents, manual compression of the uterus, and interventional or surgical procedures. A novel technique to compress the uterus by wrapping it with an elastic bandage and its outcome in 3 cases of uterine atony during cesarean section are presented. Our novel method of intermittent wrapping of the uterus during cesarean delivery seems to be a successful additional approach in the management of uterine atony during cesarean delivery and may be an alternative treatment option to other compressing procedures in order to avoid high blood loss and last but not least postpartum hysterectomy.

## 1. Introduction

Uterine atony with severe hemorrhage is a serious cause of maternal morbidity and mortality. Different methods in the management of uterine atony during cesarean delivery are well established. In addition to medical treatment by uterotonic agents, manual compression/massage of the uterus and interventional or surgical procedures are performed [1]. Those procedures include embolization of the uterine artery, uterine packing with gauze [2], uterine compression sutures [3–7], tamponade of the uterus by application of a balloon [8], bilateral arcuate artery suture [9], and ligation of the uterine artery. The aim of all these treatment options is to reduce blood loss and to avoid hysterectomy in order to sustain maternal fertility.

We developed a novel technique to compress the uterus in case of uterine atony during cesarean delivery by wrapping the uterus.

## 2. Case Presentation

We present three cases of uterine atony during cesarean delivery, which were treated with our novel technique of uterus wrapping intraoperatively. An overview of the three cases with maternal and obstetrical data is shown in Table 1. Every cesarean delivery was performed according to standard protocol in our hospital via Pfannenstiel laparotomy and transverse hysterotomy. Intraoperatively, all patients were routinely administered 10 IU of oxytocin intramyometrially to encourage uterine contraction. As uterine atony appeared, contractile agents and blood coagulation drugs (see details in Table 1) were administered according to standard protocol and uterus wrapping was performed additionally in replacement for manual compression. If the uterus was considered well contracted by clinical evaluation, the bandage was removed and the surgical procedure completed. The course of every patient was uneventful and they could leave our hospital three, four, and seven days after cesarean delivery, respectively, in good shape. The length of stay of seven days for one patient was because of nonobstetrical but neonatal reasons. Follow-up until the postpartum control six weeks after cesarean was uneventful in every case.

## 3. Technique of Uterus Wrapping

Uterus wrapping was performed in replacement for manual compression. For this purpose, the uterus was exteriorized and wrapped with a white, sterile bandage (pro-IDEAL, Promedical AG, Glarus, Switzerland; size 10 centimeters × 5 meters) concentrically from the fundus to the isthmocervical segment. In cases with long ovarian ligaments, the ovary was put aside and was not included into the wrapping. If inclusion of the ovary could not be avoided, slightly less wrapping was performed in the region of the ovary and the ovarian ligaments in order to maintain ovarian blood supply. This was also important with intention to preserve blood flow of the fallopian tubes and the infundibulopelvic ligaments. The total wrapping procedures took about 30 seconds each. If the uterus was considered well contracted by clinical evaluation (palpation of a good uterus tone and less bleeding observable), the bandage was removed and the surgical procedure completed. In case of persisting atony after removal of the bandage, the bandage was installed again until the uterine tone was assessed to be well contracted. As long as the bandage was installed, the uterus was kept exteriorized, but without tension on the parametria and adnexa. Before ending the surgical procedure of cesarean, the bandage had to be removed totally.

## 4. Case 1

A 39-year-old nulliparous woman with an uncomplicated dichorionic-diamniotic twin pregnancy was hospitalized at 40 1/7 weeks of gestation for labor induction because of the twin and postterm pregnancy. Labor was induced by insertion of a cervical ripening balloon (Cook Cervical Ripening Balloon, Cook Medical) for 24 hours, followed by 6 cycles of continuous drip of oxytocin intravenously. Each oxytocin cycle lasted for six hours and was followed by a break of two hours before starting the next cycle. Because of failure to progress in first stage of labor with a maximal cervical dilatation of 5 centimeters (cm) and insufficient contractions in the absence of oxytocin infusion, we decided to perform a cesarean delivery under spinal anesthesia at 40 5/7 weeks of gestation. Extraction of the first twin in vertex presentation was difficult because of a trapped head in the pelvis, so that the fetus had to be extracted in an inverse breech position by enlarging the hysterotomy by a T-shaped cut in caudal direction. The second fetus could easily be extracted in breech position. Each placenta was removed manually in toto. The vertical T-shaped cut of the hysterotomy was closed by single sutures and the transverse hysterotomy by continuous suturing. Because of uterine atony, uterotonic agents were administered as described in Table 1 and the uterus was exteriorized and first compressed manually. As uterotonic management by manual compression and uterotonic agents was insufficient in termination of bleeding, the uterus was wrapped as described above. The wrapping did not include the fimbriae, which allowed checking the circulation of the fallopian tubes. Uterine tone was checked regularly by palpation. After 35 minutes and 55 minutes, respectively, the bandage was removed in presumption of a good uterine tone but had to be installed again because of persistence or recurrence of atony. After a total wrapping time of 75 minutes, the bandage was finally removed. Then, the surgical procedure could be completed. Total blood loss was 2000 mL.

## 5. Case 2

**Figure 1:**
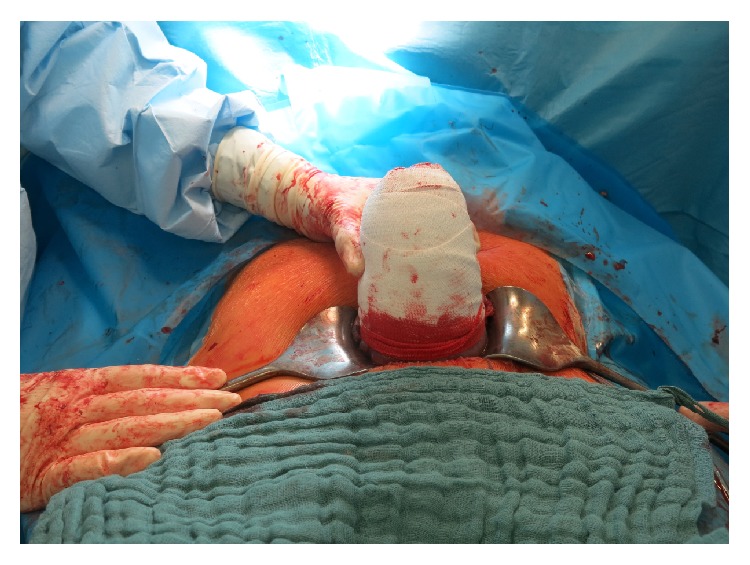
Wrapped uterus in case 2.

A 40-year-old nulliparous woman with a singleton pregnancy in vertex presentation presented herself to our obstetrical ward at 38 4/7 weeks of gestation for elective cesarean delivery because of a sonographically verified fetal malformation. Additional risk factors included gestational diabetes, treated with insulin, and a history of lumbar disc herniation. Hence, cesarean delivery was performed in general anesthesia. Because of uterine atony despite application of uterotonics as described in Table 1 and manual uterine compression, the uterus was exteriorized and wrapped as described above (Figure 1). After 18 minutes, the uterine bandage was removed, as the uterus seemed to be well contracted and really was. Then, the surgical procedure was completed. Total blood loss was 1100 mL.

## 6. Case 3

**Figure 2:**
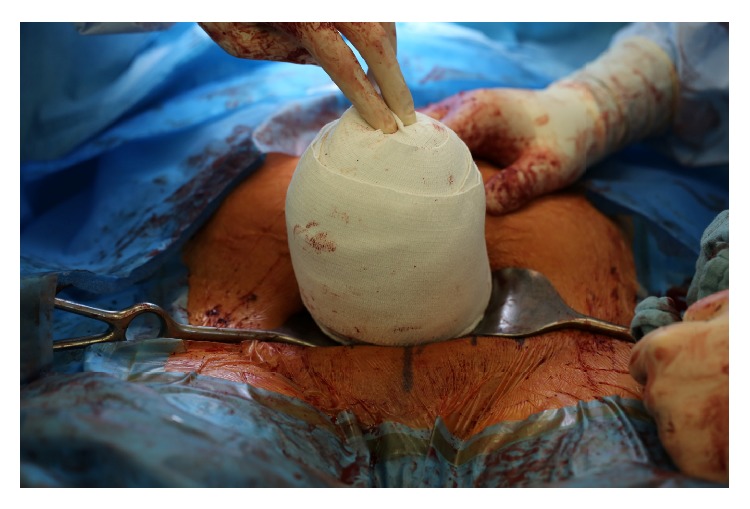
Wrapped uterus in case 3.

A 30-year-old nulliparous woman with a singleton pregnancy in vertex presentation presented herself to our obstetrical ward at 41 3/7 weeks of gestation for labor induction because of postterm pregnancy. A cesarean delivery under spinal anesthesia was performed at 42 1/7 weeks of gestation after failed induction of labor over a period of five days, including multiple oral doses of misoprostol and a cervical ripening balloon (Cook Cervical Ripening Balloon, Cook Medical) for 24 hours. Again, manual compression and uterotonics were insufficient in treatment of uterine atony. Therefore, the uterus was exteriorized and wrapped as described above (Figure 2). After 12 minutes, the uterine bandage was removed, as the uterus was well contracted. Then, the surgical procedure was completed. Total blood loss was 800 mL.

## 7. Discussion

Manual uterine compression is well established in first-line management of uterine atony, before any further procedures follow. The disadvantage of manual compression is the fact that pressure cannot be distributed equally over the whole uterus and cannot be maintained effectively and constantly over a longer period of time. Other mechanical compression procedures, as packing the uterus with gauze or tamponade of the uterus by application of a balloon, might also be less successful because of lacking counterforce from the outer face of the uterus or balloon displacement.

For this, uterus wrapping is an alternative technique for compressing the uterus. By wrapping the uterus a constant pressure can be applied to the uterus and can equally be distributed, even over a longer period of time, without causing harm to the uterus itself. As no difficult interventional or surgical procedure is necessary, it can easily and rapidly be performed when uterus atony appears, even by inexperienced surgeons. It is cheap, as only a sterile elastic bandage is needed.

Traditional interventional and surgical procedures as mentioned above could possibly cause adverse effects, such as ureteral or vascular injury, uterine synechia, myometrial necrosis, and endomyometritis [1]. But little information is given regarding subsequent fertility and pregnancy outcomes after those interventions [1].

The disadvantage of our novel method is the fact that it is sometimes difficult to decide for how long the compression by uterus wrapping has to be maintained, since a sufficient tone after removal of the bandage can worsen again. A critical point might be the problem of finding the correct amount of wrapping intensity, as the adnexa might be embedded in the wrapping and in case of too tight wrapping the blood circulation to and from the adnexa can be compromised, especially when the wrapping persists too long. In our three cases, no adverse effects appeared, even with a wrapping time of 75 minutes in one case. In one case, the bandage did not include the fimbriae, which allowed checking the circulation of the fallopian tubes.

We conclude that our novel method of wrapping the uterus seems to be a successful way of continuously compressing the uterus over a longer time during cesarean delivery in case of uterine atony. We speculate that it is able to minimize blood loss during cesarean delivery, if applied early, and may substitute more invasive operative procedures.

## Figures and Tables

**Table 1:** 

Case	Age (years)	Parity	Gestational age (weeks)	Duration of cesarean section (min)	Duration of uterus wrapping (min)	Blood loss (mL)	Preoperative hemoglobin (g/L)	Post operative hemoglobin (g/L)	Medication
1	39	1	40 5/7	93	75	2000	125	97	200 mcg misoprostol sublingually, 20 IU oxytocin i.v., 500 mcg sulprostone i.v., 1 g tranexamic acid i.v., 1 g calcium i.v., and 1250 IU factor XIII i.v.

2	40	1	38 4/7	55	18	1100	112	90	400 mcg misoprostol sublingually, 10 IU oxytocin i.v., 500 mcg sulproston intramyometrially, 1000 mcg sulprostone i.v., 1 g tranexamic acid i.v., 1 g calcium i.v., and 1250 IU factor XIII i.v.

3	30	1	42 1/7	58	12	800	134	121	400 mcg misoprostol sublingually and 20 IU oxytocin i.v.
